# FDBRP: A Data–Model Co-Optimization Framework Towards Higher-Accuracy Bearing RUL Prediction

**DOI:** 10.3390/s25175347

**Published:** 2025-08-28

**Authors:** Muyu Lin, Qing Ye, Shiyue Na, Dongmei Qin, Xiaoyu Gao, Qiang Liu

**Affiliations:** 1 School of Computer Science, Yangtze University, Jingzhou 434023, China; 2023710712@yangtzeu.edu.cn (M.L.); 2023710708@yangtzeu.edu.cn (S.N.); 2023710709@yangtzeu.edu.cn (D.Q.); 2023710706@yangtzeu.edu.cn (X.G.); 2023710714@yangtzeu.edu.cn (Q.L.); 2 Artificial Intelligence Research Platform, Yangtze University, Jingzhou 434023, China

**Keywords:** remaining useful life prediction of bearings, multi-scale spatio-temporal modeling, data augmentation, prognostic and health management

## Abstract

**Highlights:**

- This study applies sliding-window data augmentation, followed by biaxial signal concatenation and lifespan label normalization to enhance signal representation.
- Preprocessed data are input into a feature extraction module that combines an enhanced graph convolutional network for spatial modeling and a multi-scale temporal convolutional network, with an attention mechanism optimizing feature consistency to capture degradation.
- The model enhances receptive field coverage with optimized dilated convolution and residual connections. Comparative experiments and ablation studies validate its effectiveness and generalization.

**Abstract:**

This paper proposes Feature fusion and Dilated causal convolution model for Bearing Remaining useful life Prediction (FDBRP), an integrated framework for accurate Remaining Useful Life (RUL) prediction of rolling bearings that combines three key innovations: (1) a data augmentation module employing sliding-window processing and two-dimensional feature concatenation with label normalization to enhance signal representation and improve model generalizability, (2) a feature fusion module incorporating an enhanced graph convolutional network for spatial modeling, an improved multi-scale temporal convolution for dynamic pattern extraction, and an efficient multi-scale attention mechanism to optimize spatiotemporal feature consistency, and (3) an optimized dilated convolution module utilizing interval sampling to expand the receptive field, and combines the residual connection structure to realize the regularization of the neural network and enhance the ability of the model to capture long-range dependencies. Experimental validation showcases the effectiveness of proposed approach, achieving a high average score of 0.756564 and demonstrating a lower average error of 10.903656 in RUL prediction for test bearings compared to state-of-the-art benchmarks. This highlights the superior RUL prediction capability of the proposed methodology.

## 1. Introduction

With the rapid advancement of manufacturing technologies and industrial automation, Prognostics and Health Management (PHM) has become increasingly critical for enhancing system reliability and operational efficiency across various industries. As an integrated approach, PHM typically encompasses several key components: health condition monitoring, fault diagnosis and prognosis, remaining useful life prediction, health management, and maintenance decision-making [1]. Among these, RUL prediction serves as a fundamental module that not only provides valuable insights into system operational availability but also effectively mitigates potential safety hazards and economic losses caused by unexpected equipment failures during normal operation [2].

In modern production and research systems, mechanical components serve as fundamental elements, among which rolling bearings constitute one of the most critical structural parts. Statistical evidence indicates that approximately 30% of rotating machinery failure cases result from bearing degradation or failure [3]. Consequently, accurate prediction of bearing RUL has emerged as a pressing research challenge, aiming to substantially mitigate adverse consequences including unexpected system failures, increased manufacturing costs, reduced production efficiency, and potential safety risks.

Current methodologies for predicting bearing Remaining Useful Life can be primarily categorized into two approaches: model-driven methods and data-driven methods [4].

Model-driven approaches for bearing RUL prediction involve constructing mathematical representations of physical or empirical models to characterize bearing degradation mechanisms, typically formulated through a series of mathematical equations [5,6,7].

Huang et al. [8] proposed the Empirical Mode Decomposition (EMD) algorithm, which effectively processes non-stationary signals but suffers from limitations such as mode mixing and end effects. To address these issues, Gilles et al. [9] introduced the Empirical Wavelet Transform (EWT), enabling adaptive frequency band segmentation via wavelet filter banks for signal decomposition. While EWT adaptively extracts subtle fault characteristics, it encounters challenges in noise suppression, computational efficiency, and generalizability. Dragomiretskiy and Zosso [10] developed the Variational Mode Decomposition (VMD) algorithm, which effectively isolates multi-frequency fault features. However, VMD exhibits high computational complexity and parameter sensitivity, requiring meticulous tuning. Jiang et al. [11] subsequently improved VMD by integrating an initial center frequency-guided approach with intelligent optimization algorithms, thereby resolving its parameter adjustment limitations.

While model-driven methods can achieve accurate RUL prediction for specific bearings through customized modeling, their effectiveness remains constrained to particular bearing types. These approaches exhibit strong dependence on expert knowledge for model formulation, limiting their generalizability across diverse mechanical systems. Furthermore, they demonstrate weak robustness when operating under complex working conditions.

In contrast, data-driven methodologies analyze historical operational data collected from bearing monitoring equipment to establish degradation-representative health indicators (HIs) and predictive models. By leveraging statistical theory and machine learning techniques, these approaches enable direct RUL prediction for rolling bearings without requiring explicit physical modeling [12,13].

Ahmed, A. et al. [14] introduce a taxonomy of feature learning methods and also discuss methods of feature-learning under imbalanced data samples and different operational settings to assist scholars and practitioners in understanding this area. Zhang Wenlong [15] achieved bearing RUL prediction by analyzing monitoring data and extracting degradation-representative features as predictive covariates. Wu Zexun [16] employed Convolutional Neural Networks (CNNs), utilizing convolutional kernels to extract features from historical operational data, attaining satisfactory prediction accuracy. Chen Changchuan et al. [17] enhanced traditional CNNs to Fully Convolutional Networks (FCNs), leveraging their self-learning capability for autonomous feature extraction in RUL prediction. Bao Wenxia et al. [18] proposed a Double-CNN architecture with parallel convolutional channels, demonstrating improved RUL prediction performance through dual-path feature learning. While CNN-based methods show promise, they frequently encounter gradient vanishing issues during training, leading to significant prediction deviations.

Xu Zili [19] adopted Recurrent Neural Networks (RNNs) to capture temporal dependencies in bearing degradation data. Compared to CNNs, RNNs demonstrate superior performance for time-series RUL prediction. However, RNNs suffer from the “vanishing gradient” problem, where layers receiving minor gradient updates cease learning, leading to compromised long-sequence feature retention. Long Short-Term Memory (LSTM) networks address this limitation through gating mechanisms, significantly enhancing long-term temporal feature learning and prediction accuracy. Kamat, P. et al. [20] investigated RUL of bearings with an enhanced feature selection strategy and anomaly monitoring of bearing operational data, using different LSTMs to capture complex temporal dependencies and spatial correlations in the bearing sensor data. Sahu, P.K. et al. [21] proposed a bearing RUL prediction method by combining an absolute cumulative modified multiscale permutation entropy HI with an LSTM deep learning model. Sun Qi et al. [22] developed a hybrid CNN-LSTM architecture that concurrently processes short-term degradation patterns and preserves long-term temporal influences. Gu Yingkui et al. [23] implemented Bidirectional LSTM (Bi-LSTM) with AdaMax optimization for adaptive hyperparameter tuning, mitigating gradient vanishing issues inherent in RNNs. Despite their advantages, LSTMs exhibit high computational complexity, excessive parameter counts, and prolonged inference times, limiting their industrial deployment.

The Gated Recurrent Unit (GRU) represents a streamlined variant of LSTM architecture, retaining comparable advantages in temporal modeling while featuring a simplified structure. This efficiency has led to its widespread adoption in RUL prediction applications. Xiao Li [24] integrated GRUs with particle filtering to precisely track bearing degradation processes, demonstrating enhanced RUL prediction accuracy. Han Linjie [25] developed a CNN-GRU hybrid framework (GRU-HI model) to assess bearing degradation trends, capitalizing on GRU’s temporal processing strengths to generate lifespan degradation curves. Ye Linfeng et al. [26] extended the architecture through Bidirectional GRU (Bi-GRU), employing dual GRUs to simultaneously process forward and backward temporal dependencies, thereby improving model learning capacity for lithium-ion battery RUL prediction. Despite these advancements, GRU-based approaches exhibit suboptimal long-sequence modeling capability, constrained local feature extraction performance, reduced parallelization potential due to sequential dependencies, and training efficiency requiring further optimization.

While most current bearing RUL prediction research favors RNN variants for temporal data processing, Bai et al. [27] argue that this perspective is outdated. Their empirical evaluations demonstrate that convolutional networks should be considered primary candidates for sequence modeling, as they often outperform RNN-based architectures while circumventing recurrent models’ inherent limitations, such as gradient explosion/vanishing issues and memory retention constraints. Wu Shuping [28] introduced Residual Connections (ResNet) to enhance traditional CNNs, achieving higher prediction accuracy through improved gradient flow. Wang Shuai et al. [29] developed an enhanced Temporal Convolutional Network (TCN), incorporating Multi-head Attention (MA), which processes multiple attention mechanisms in parallel to boost feature extraction efficiency. Gao Meng et al. [30] proposed a hybrid TCN-BiLSTM framework with a Convolutional Attention Module (CAM) to expand the network’s receptive field and address long-term dependencies, yielding superior prediction precision. Notwithstanding these advances, TCN architectures continue to grapple with two principal limitations: substantial memory requirements due to intermediate feature storage and vulnerability to local noise perturbations, both of which adversely impact predictive reliability.

In summary, to achieve improved spatiotemporal feature extraction, long-sequence modeling, and feature representation capabilities while enhancing generalization and inference efficiency, this paper proposes a convolutional neural network-based approach incorporating Deep Residual Networks (DRNs) with shortcut connections (SC) to address performance degradation in deep networks through identity mapping, along with Temporal Convolutional Networks that utilize fully convolutional architectures and dilated causal convolutions to accurately capture temporal degradation characteristics from historical data. The effectiveness and superiority of this method are validated through analysis of bearing degradation datasets.

Compared with the already known hybrids ResNet + TCN + attention [31], the proposed method overcomes the inherent spatiotemporal feature fragmentation by synergistically integrating dilated causal convolutions for long-term temporal dependencies and self-attention graph convolutions for spatial sensor topology modeling. An innovative multi-scale parallel architecture enhances feature expressiveness—the refined temporal convolution captures different fault patterns, while adaptive attention mechanisms suppress noise interference. The modular lightweight design enables GPU-accelerated computation, demonstrating on the XJTU-SY dataset superior accuracy, enhanced robustness, and deployment readiness for industrial predictive maintenance.

The contributions of this paper are summarized as follows:

(1) The Data Augmentation Module (DAM) employs sliding window techniques to expand dataset features and further concatenates two-dimensional features, facilitating the capture of more degradation patterns. The Dilated Causal Module (DCM) not only captures long-term temporal dependencies but also maintains the causal properties of time-series data, addressing limitations in long-sequence modeling found in previous approaches.
(2) The Feature Fusion Module (FFM) utilizes self-attention-based graph convolution for spatial modeling to more effectively capture spatial dependencies in bearing vibration signals, while employing an improved multi-scale temporal convolution module to extract temporal features, alleviating previous limitations in temporal modeling. The efficient multi-scale attention module enhances feature consistency and expressiveness through global adaptive pooling and SoftMax weighting mechanisms, reducing shortcomings in feature fusion found in prior methods. The combined convolutional blocks improve computational efficiency, reduce temporal dependencies in the model, and enable higher efficiency through parallel computing. The multi-scale temporal convolutional network and attention mechanisms effectively filter noise, improving robustness against noise and outliers.
(3) Using the XJTU-SY bearing full-life test dataset, comparative experiments with other advanced models and ablation studies of the proposed method itself demonstrate through multiple evaluation metrics that our approach achieves smaller prediction errors and higher accuracy.

The subsequent sections are organized as follows: Section 2 presents the theoretical background, Section 3 describes the proposed theoretical modules and bearing RUL prediction model, Section 4 validates the effectiveness of the proposed method through comparative experiments and ablation studies on the XJTU-SY dataset, and finally Section 5 concludes the paper.

## 2. Theoretical Foundations

### 2.1. Deep Residual Networks

Deep Residual Networks represent an advanced deep neural network architecture developed from classical convolutional neural networks. By introducing a “skip connection” structure, DRN effectively addresses the gradient vanishing and training degradation problems inherent in conventional CNNs.

**Figure 1:**
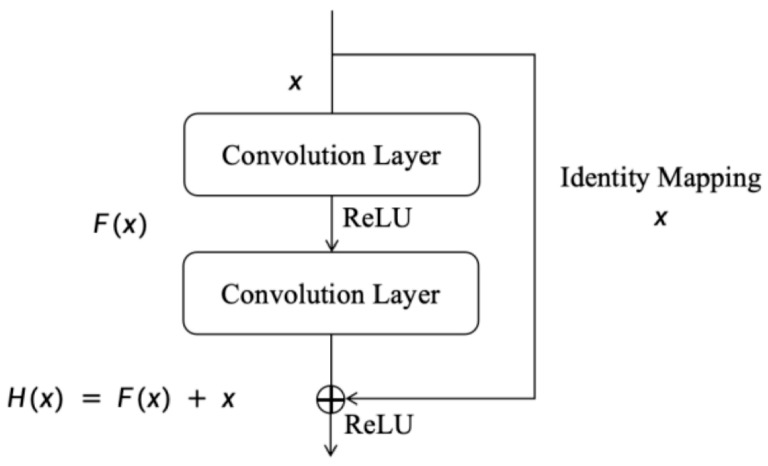
Basic Residual Unit.

The fundamental residual unit of a deep residual network is illustrated in Figure 1.

The basic residual unit consists of: x-input, $H\left(x\right)$-output, $F\left(x\right)$-residual mapping function.

Assuming a DRN is composed of L sets of fundamental residual units, where each unit’s input, output and parameters are denoted as $x_{l}$, $x_{l+1}$ and $W_{l}$ respectively, we obtain:

(1) $$y_{l}=h\left(x_{l}\right)+F(x_{l},\;W_{l})$$

(2) $$x_{l+1}=f(y_{l})$$

In the equation: F(·)-residual function, f(·)-ReLU function.

When the identity mapping is optimal (i.e., $h\left(x_{l}\right)=\;x_{l}$, $f\left(y_{l}\right)=\;y_{l}$), Equation (2) can be transformed into:

(3) $$x_{l+1}=x_{l}+F(x_{l},\;W_{l})$$

Then through recursive iteration, the output of the L−th residual block can be derived as:

(4) $$x_{L}=x_{l}+\;\sum_{i=1}^{L-1}F(x_{l},\;W_{l})$$

### 2.2. Temporal Convolutional Network

The Temporal Convolutional Network is a convolutional network architecture designed for time series processing, built upon residual networks. TCN primarily addresses the insufficient generalization capability of LSTM-type networks [32]. As an alternative model that can potentially surpass traditional recurrent networks, TCN exhibits two distinctive advantages: First, it employs combined dilated convolutions with residual convolutions to ensure both the integrity of temporal information transmission and the effectiveness of feature extraction. Second, through proper configuration of convolution kernels, pooling, and stride, one-dimensional convolution can achieve sequence-length flexibility similar to RNNs when parameters are appropriately set—meaning it imposes no constraints on output sequence length.

Given an input sequence $x_{0},\;x_{1},\;\ldots ,\;x_{t}$, where we aim to predict corresponding outputs $y_{0},\;y_{1},\;\ldots ,\;y_{t}$ at each time step, with the constraint that outputs can only be derived from historical data prior to the current t−1 timestep, a standard TCN mathematical model can be expressed as:

(5) $$\left(y_{0},\;y_{1},\;\ldots ,\;y_{t}\right)\;=\;f_{TCN}(x_{0},\;x_{1},\;\ldots ,\;x_{t})$$

where $f_{TCN}(\cdot )$-represents the ordered connection between convolutional networks and feedforward networks.

In TCN, two commonly used convolution methods are dilated convolution and residual convolution, whose mathematical models are shown in Equations (6) and (7), respectively.

Dilated Convolution:

(6) $$F\left(i\right)=\sum_{j=0}^{k-1}h(j)x(i-dj)$$

where k-side length of convolution kernel, h-convolution kernel function, i-element index in sequence, d-dilation factor, d<k.

Residual Convolution:

(7) $$o=f_{activation}(x+f_{res}(x))$$

where $f_{activation}(\cdot )$-network activation function, $f_{res}(\cdot )$-residual function.

## 3. Proposed Algorithm

### 3.1. Data Augmentation Module

**Figure 2:**
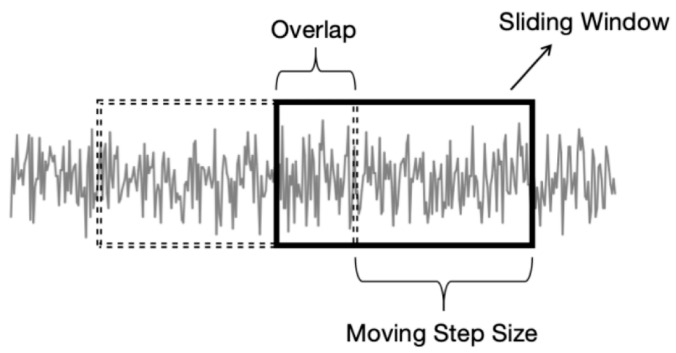
Schematic Diagram of Sliding Window Method.

To enhance dataset feature diversity and improve the neural network’s generalization capability, we design the Data Augmentation Module. The module first employs an overlapping sliding-window sampling method for effective data augmentation, as illustrated in Figure 2:

DAM sets the sliding window length to 3 with a step size of 2, achieving a 33.3% overlap rate. Taking Bearing1_1 as an example, the original dataset contains 4,030,464×1 sample sequences, which increases to 6,045,694×1 after resampling.

The resampled data undergoes further processing where both vertical and horizontal signals are reshaped into (−1,  32,768) matrices and concatenated into (−1,  32,768,  2) feature maps to comprehensively consider vibration signals from multiple sensors for better feature extraction in life prediction. Following an end-to-end life prediction approach, we add life labels to the concatenated dataset and normalize the full-life degradation data of bearings using the formula:

(8) $${Lable}_{i}=\frac{S-i}{S}$$

where *S*—total sample count, *i*—time point, ${Lable}_{i}$—life label at the *i*th time point.

The normalized bearing life data is mapped between 0 and 1, when the life label ${RulT}_{i}=1$ indicates a brand-new bearing and when the life label ${RulT}_{i}=0$ represents the bearing has completely failed, that is, the remaining service life has been exhausted.

### 3.2. Feature Fusion Module

**Figure 3:**
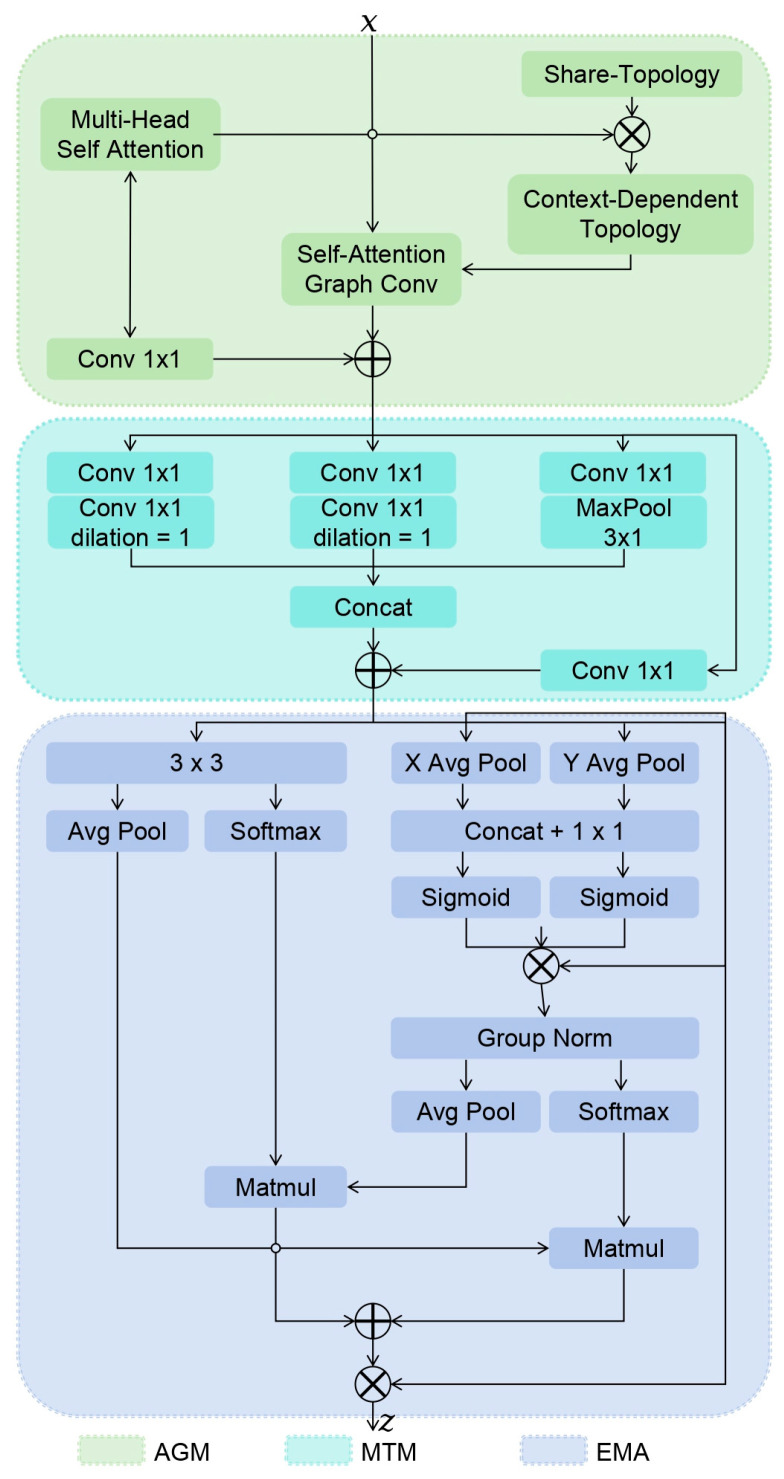
Spatio-temporal Feature Fusion Module.

This paper designs a novel lightweight spatiotemporal feature fusion module (FFM) to provide rich feature representations for subsequent operations. As shown in Figure 3, the FFM consists of three core submodules: the Self-attention-graph Convolution module (AGM) for spatial modeling, the Multi-scale Time Convolution Module (MTM) for temporal modeling, and the Efficient Multi-scale Attention module (EMA).

Herein, the AGM employs self-attention mechanisms on joint features to deduce intrinsic topological relationships, which are subsequently incorporated as neighborhood vertex information for Graph Convolution operations [33]. While AGM effectively captures local structural relationships in skeletal connections through its attention mechanism, this localized focus may potentially overlook broader contextual information.

Concurrently, the MTM extracts hierarchical temporal features through parallel convolutional branches featuring distinct kernel sizes and dilation rates [34]. Although MTM demonstrates competent multi-scale feature extraction capability, its effectiveness in comprehensively capturing all critical features across varying temporal scales may be situationally constrained.

To address these limitations, we introduce the EMA module that performs adaptive feature processing and weighting through a series of operations including global adaptive pooling, 1×1 or 3×3 convolutions, and SoftMax weighting mechanisms. The EMA first integrates intra-channel features via 1×1 and 3×3 convolutions, then applies global adaptive pooling and SoftMax weighting to generate attention-enhanced feature representations. This processing enhances both global-local feature consistency and expressive power through channel-wise operations, complementing AGM’s local relationship modeling and MTM’s multi-scale feature extraction.

The complete FFM architecture combines these three specialized submodules in a computationally efficient design: AGM is responsible for spatial modeling, and the self-attention mechanism is used to infer the intrinsic topology of skeletal connections, which reduces the dependence on predefined adjacency matrix in traditional graph convolution and reduces the computational complexity, MTM is responsible for temporal modeling and uses multi-branch convolution operation to extract multi-scale temporal features through different kernel sizes and dilation rates, avoiding the limitation of a single convolution kernel and reducing the number of parameters at the same time, EMA is responsible for feature weighting, and the channel features are fused by 1×1 and 3×3 convolution, and the global adaptive pooling and SoftMax weighting mechanism are combined to realize the adaptive weighting processing of features, reduce the size of the feature map, reduce the amount of subsequent calculation, and retain important global information. This design not only reduces the number of parameters but also enhances the expression ability of features and improves the computational efficiency.

### 3.3. Dilated Causal Convolution Module

**Figure 4:**
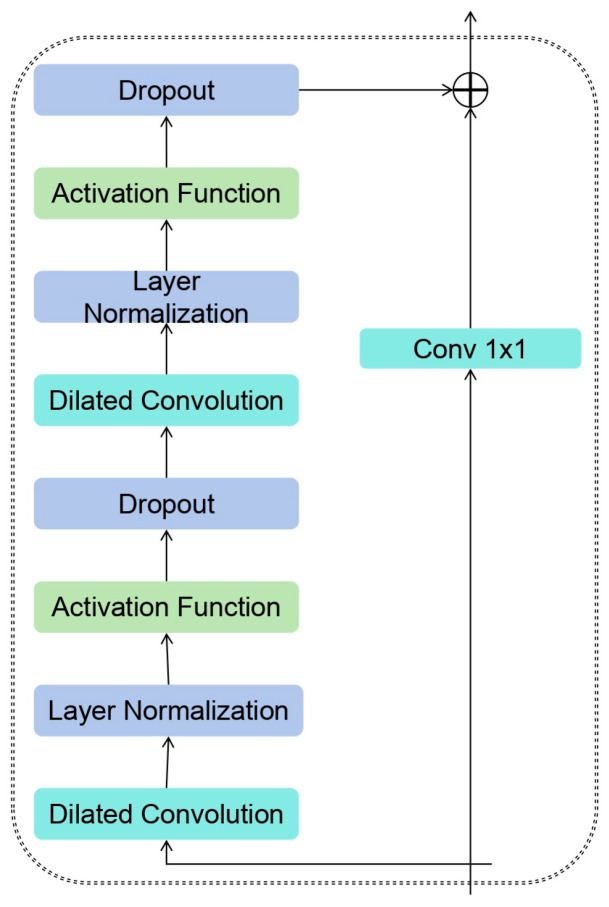
Residual Structure of TCN.

This paper proposes a novel Dilated Causal Convolution Module incorporating Multiple Parallel Temporal Convolutional Networks (MPTCN) to extract local features from bearing vibration signals at current time scales. Its basic residual block structure is shown in Figure 4, and for a one-dimensional sequence input $g=\{s_{1},\;s_{2},\;\ldots \;s_{n-1}\}\in R^{n}$ and a convolution kernel f:{0, 1, …, z−1}→R, the extended convolution operation F of a sequence element S is defined as in Equation (9).

(9) Fs=g·fds=∑i=0z−1fi·gs−di

**Figure 5:**
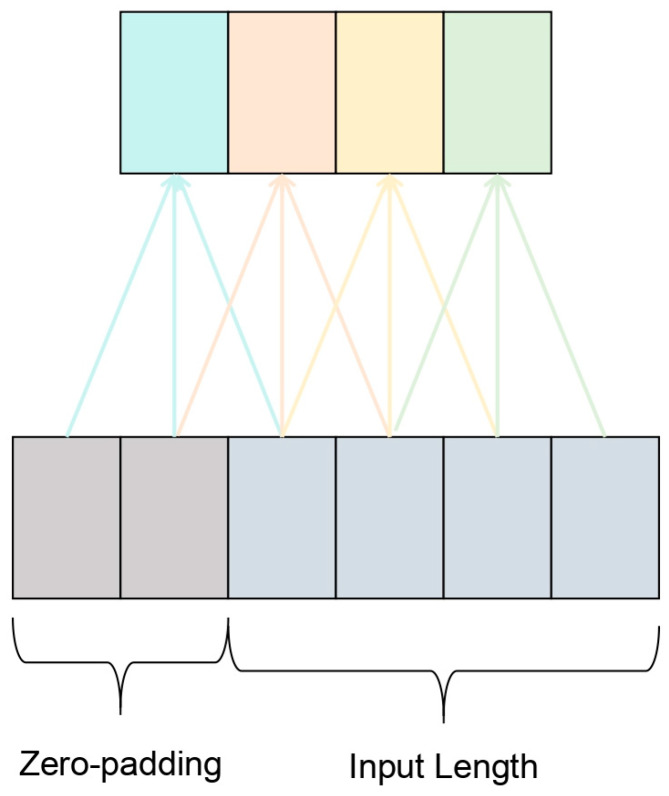
Zero-padding Convolutional Structure.

TCN is based on two principles: the fact that the network generates an output of the same length as the input, and the fact that it has never come back to the past and there could be no leakage. To complete the first point, TCN uses a one-dimensional Full Convolutional Neural Network architecture, in which each hidden layer has the same length as the input layer and a corresponding length of zero padding is added to keep the subsequent layers of the same length as the previous layers, as shown in Figure 5. To achieve the second point, TCN uses causal convolution, where the output at time t is convolution only with time t and the earlier elements in the previous layer.

**Figure 6:**
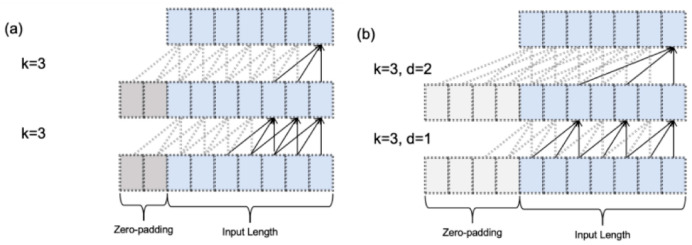
(**a**) Causal Convolution without Expansion Factor; (**b**) Causal Convolution with Expansion Factor.

While causal convolution preserves temporal dependencies in vibration data, its receptive field remains constrained by kernel size (Figure 6a), traditionally requiring deeper networks for longer dependencies. The introduced dilation factor (Figure 6b) solves this by exponentially expanding receptive fields without network deepening. Formally defined in Equation (9), the dilation factor determines the step interval between input elements used for output computation, enabling efficient long-range pattern capture.

The introduction of causal convolutions allows the neural network to take into account the temporal order dependencies present in bearing vibration data, that is, causal associations in time. However, similar to traditional convolutional neural networks, causal convolutions are also limited by the size of the convolution kernel, which limits the time steps that can be considered for the output elements, as shown in Figure 6a. To capture longer dependencies, it is often necessary to build deeper networks. Therefore, the concept of inflation factor is introduced to increase the utilization of more historical data before the current moment in the output part while keeping the network shallow; the structure is shown in Figure 6b. The inflation factor represents the distance between the elements of the input sequence, which are used to calculate the output result, and the calculation is defined as in Equation (9).

Therefore, the conventional convolutional layer can be regarded as an extended convolutional layer with a dilation factor of 1, given that the input terms for the output values are contiguous. When analyzing time series data, the convolution kernel size and dilation factor can be analogized to sampling points and sampling intervals, respectively. For the same number of sampling points, a longer sampling interval encompasses richer temporal information. Utilizing the dilation factor enables the network to achieve a broader time span without incurring the information loss typically associated with down sampling. This is because, unlike down sampling, dilated convolution does not alter the length of the output features. As depicted in Figure 6b dilated convolution expands the receptive field through interval-based sampling, enabling the network to revert to $1+\sum_{i=0}^{n-1}(k-1)d_{i}$ time steps, where k represents the convolution kernel size, d denotes the dilation factor, and n signifies the number of layers beneath the current layer. It is evident that, with an identical number of layers, the output in Figure 6b captures more comprehensive time series information compared to Figure 6a.

**Figure 7:**
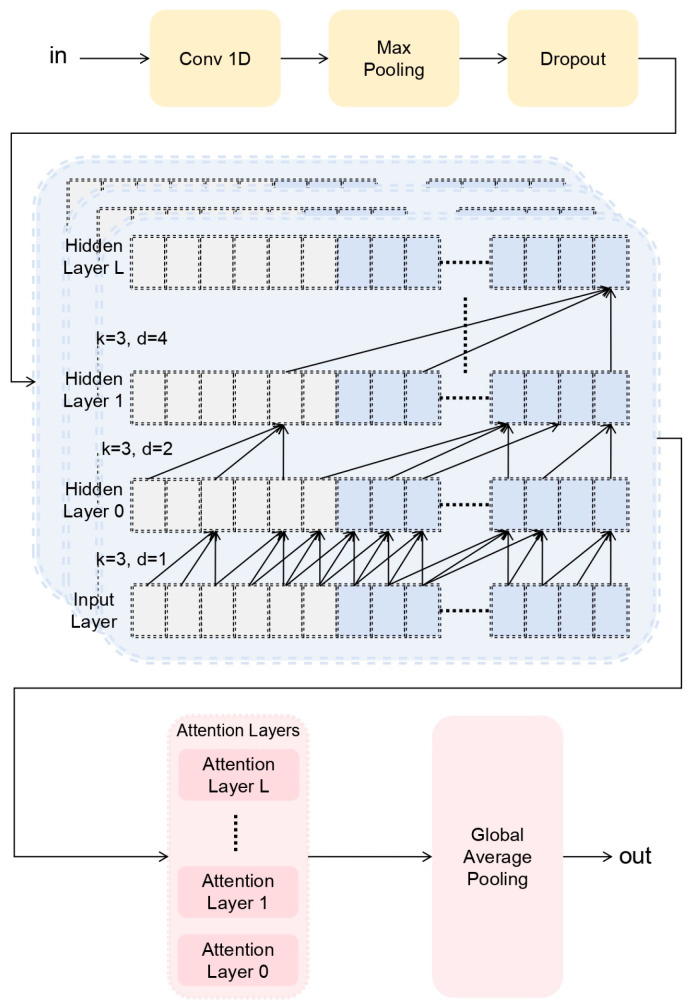
MPTCN Structure in Dilated Causal Convolution Module.

Compared with RNNs, the dilated causal convolutional module offers significant advantages through weight sharing and local perception in convolutional layers. Weight parameter sharing effectively reduces the number of trainable parameters in the network, while locally aware features accurately capture the structural information of the input data covered by the current convolution kernel. Consequently, DCM not only learns long-term temporal correlations in input time series but also enables parallel computation akin to CNNs. Although RNNs theoretically possess the ability to capture infinitely long histories, the Temporal Convolutional Networks within DCM have proven more suitable for domains requiring long-term historical dependencies, as demonstrated in Figure 7 for the MPTCN structure.

In addition, DCM also contains residual connections, which enables the structure to obtain stable deep networks. The residual block consists of two convolutional layers and the Gaussian error linear unit activation function GELU, and batch normalization is added to each layer to regularize the network and enhance its generalization ability.

The algorithm flow of DCM is briefly summarized as follows (see Algorithm 1):
**Algorithm 1:** Flow of DCMInput: $\mathrm{sequence}\;\mathrm{input}:\;g=\{s_{1},s_{2},\ldots s_{n-1}\}\in R^{n}$, convolution kernel f:{0,1,…,z−1}→R Procedure: P1: dilated convolution: Fs=g·fds=∑i=0z−1fi·gs−di P2: $\mathrm{causal}\;\mathrm{convolution}:\;y_{t}=\sum_{k=0}^{min(t,K-1)}w_{k}\cdot x_{t-k}$ P3: $\mathrm{TCN}\;\mathrm{layer}\;\mathrm{expansion}\;\mathrm{rate}:\;{expansion\_rate}_{i}=2^{i-1}$ P4: $\mathrm{Receptive}\;\mathrm{field}\;\mathrm{expansion}:\;time_{step}=1+\sum_{i=0}^{n-1}(k-1)d_{i}$ Output: $\mathrm{RUL}\;\mathrm{predictor}:\;y=MPTCN\left(x\right)⨁RES(x)$


### 3.4. Bearing Remaining Service Life Prediction Model

**Figure 8:**
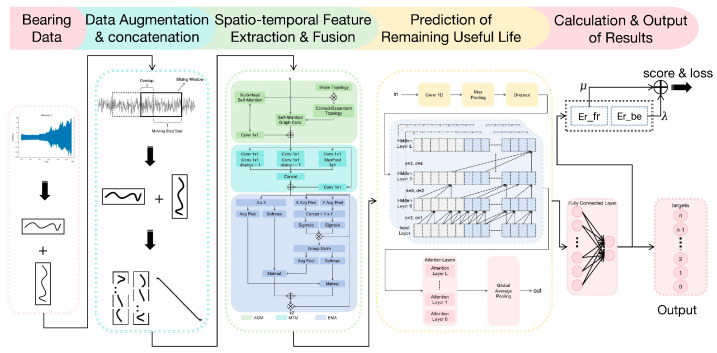
Schematic Diagram of FDBRP.

The proposed prediction model, Feature fusion and Dilated causal convolution model for Bearing Remaining useful life Prediction (FDBRP), is illustrated in Figure 8 with a schematic diagram of its structure. The overall structure of the network is mainly composed of DAM, FFM and DCM.

For bearing vibration signals, the process begins with the application of the sliding window method within DAM to augment the dataset’s feature quantity. This step involves concatenating features across two dimensions to enhance the generalization capability of the neural network model. Subsequently, in the spatio-temporal FFM, spatial modeling is achieved through a graph convolution module based on self-attention mechanisms, while temporal modeling is conducted using a multi-scale temporal convolution module. These processes independently extract spatial and temporal features, which are then fused via an efficient multi-scale attention mechanism. Specifically, global adaptive pooling and a SoftMax weighting mechanism are employed to assign channel-wise weights to the extracted features, thereby generating an attention-based fusion representation that improves the consistency and expressiveness of spatio-temporal features. DCM ensures that the model’s output at each time point depends solely on the current and preceding inputs, adhering to the inherent characteristics of time-series data. Additionally, dilated convolution enables the model to capture long-range dependencies without increasing its parameter count by strategically skipping certain inputs during the convolution operation. Finally, the remaining service life of the bearing is predicted based on the aforementioned processes.

## 4. Experimental Results and Analysis

### 4.1. Data Sources

The experimental data in this study were obtained from the XJTU-SY rolling bearing accelerated life test dataset, which was collaboratively developed by Professor Yaguo Lei’s research team at the School of Mechanical Engineering, Xi′an Jiaotong University, and SUMYOUNG TECH (Changxing Shengyang Technology Co., Ltd., Huzhou, China) through a two-year accelerated life testing program. This comprehensive dataset contains complete life cycle vibration signals of 15 rolling bearings under three different operating conditions, characterized by its high sampling frequency, substantial data volume, diverse failure modes including spalling, cracking and wear, as well as meticulously recorded metadata [35].

**Figure 9:**
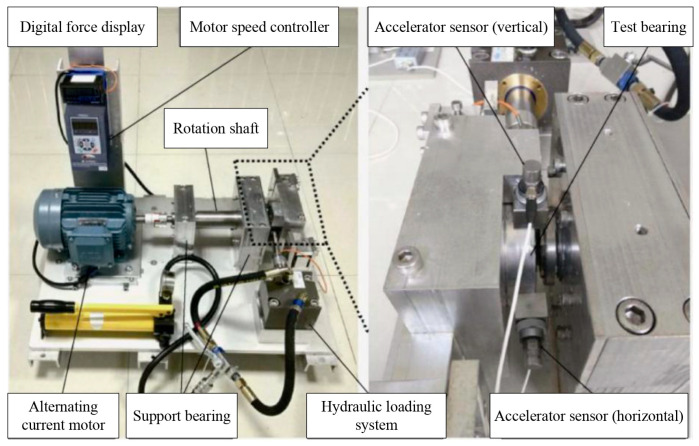
Bearing life degradation test bench.

The bearing life degradation test platform, shown in Figure 9, consists of an AC motor with speed control system, rotating shaft, support bearings, hydraulic loading system, and test bearings for data acquisition.

The test bearing is LDK UER204 rolling bearing, and its relevant parameters are shown in Table 1.

**Figure 10:**
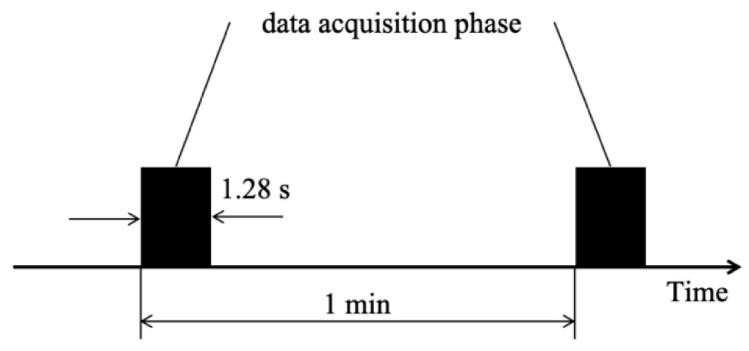
Bearing vibration signal sampling Settings.

The experimental platform is equipped with PCB 352C33 unidirectional acceleration sensors in the horizontal and vertical directions to obtain the life cycle degradation data of the bearing, and the DT9837 portable dynamic signal acquisition device is used to collect the vibration signal of the bearing in the acceleration experiment process. The sampling frequency is 25.6 kHz, the sampling interval is 1 min, and each sampling market is 1.28 s. The bearing vibration signal sampling setting is shown in Figure 10.

**Figure 11:**
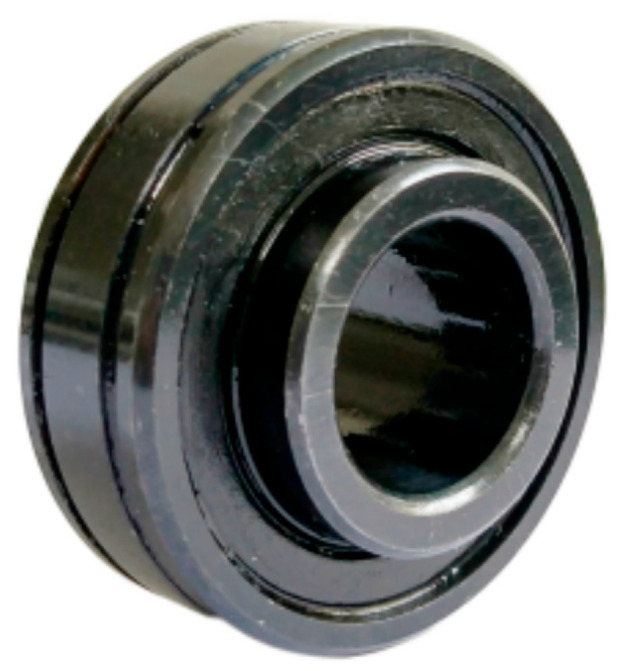
Basic structure of LDK UER204 rolling bearing.

Figure 11 shows the basic structure of LDK UER204 rolling bearing.

To ensure the validity of experimental comparisons for bearing RUL prediction, this study consistently utilizes data obtained under the operating condition of 2100 rpm rotational speed with a 12 kN radial load. The following hypotheses hold for the dataset: (1) All experimental data are valid and reflect true measurements. (2) The experimental environment is strictly maintained throughout each test.

The multiple bearing datasets acquired under this identical operating condition through the DAM are vertically concatenated (v-stack operation) to facilitate cross-domain generalization prediction. The detailed data processing methodology is presented in Table 2.

### 4.2. Evaluation Indicators

To evaluate the performance of the proposed algorithm, in this paper, the Mean Absolute Error (MAE), Root Mean Square Error (RMSE), Mean Absolute Error in the second half of the stage (MAE_SH), Root Mean Square Error in the second half of the stage (RMSE_SH) and Score were used as evaluation indicators to evaluate the model. The mathematical formulations are

(10) $$MAE=\frac{1}{n}\sum_{i=1}^{n}\left|\hat{y_{i}}-y_{i}\right|$$

(11) $$RMSE=\sqrt{\frac{1}{n}\sum_{i=1}^{n}w_{i}{\left(\hat{y_{i}}-y_{i}\right)}^{2}}$$

(12) $$MAE\_SH=\frac{1}{n}\sum_{i=1}^{n}\left|\hat{y_{i}}-y_{i}\right|\;\;\;(m\geq inx\_mid)$$

(13) $$RMSE_{SH}=\sqrt{\frac{1}{n}\sum_{i=1}^{n}w_{i}{\left(\hat{y_{i}}-y_{i}\right)}^{2}}\;\;\;(m\geq inx\_mid)$$

where $\hat{y_{i}}$ is the RUL predicted by the model at the ith time point, y is the true RUL at the *i*th time point, *n* is the size of the data point, and m is the size of the last half of the data points.

(14) $$Score=0.35\times mean\left(score\_sh\right)+0.65\times mean(score\_sh)$$

Here, score_fh represents the early-stage prediction score and score_sh corresponds to the late-stage prediction score. In prognostic analysis, prediction errors during the late degradation phase are considerably more critical than those in the early phase. This is because prediction inaccuracies during healthy operational periods have minimal practical consequences for production and maintenance planning, whereas prediction errors approaching failure conditions can significantly impact operational safety and equipment reliability. Consequently, the Score formulation assigns different weighting factors to these two phases. Additionally, the computational approach varies depending on the specific relationship between the model’s predicted RUL and the actual RUL value at each evaluation point, as detailed below:

(15) $$Score=0.35\times mean\left(score_{fh}\right)+0.65\times mean(score\_sh){Lable}_{i}=\frac{S-i}{S}$$

(16) $$score_{2}=e^{-\ln\left(0.6\right)\times \frac{\hat{y_{i}}-y_{i}}{10}}\;\;\;(\hat{y_{i}}\geq y_{i})$$

The evaluation metrics are defined as follows: Er_1_ represents the arithmetic mean of MAE and RMSE for early-to-mid stage predictions, while Er_2_ denotes the corresponding average of MAE_SH and RMSE_SH specifically for the critical late-stage predictions. Recognizing that prediction accuracy near the failure threshold carries greater operational significance in bearing life assessment, Er_A_ is calculated as a weighted average with higher importance of Er_2_, with the specific weighting formulation expressed as:

(17) $$Er_{1}=\frac{MAE+RMSE}{2}$$

(18) $$Er_{2}=\frac{MAE\_SH+RMSE\_SH}{2}$$

(19) $${Er}_{A}=0.4\times Er_{1}+0.6\times Er_{2}$$

### 4.3. Experimental Verification

To validate the effectiveness of the proposed feature extraction module and the prediction model’s accuracy, this study conducts comparative RUL prediction tests on Ds2 using five feature extractors: (1) the proposed FFM, (2) conventional CNN, (3) Inception-ResNet for multi-scale feature extraction, (4) Channel Attention with dynamic channel-weighting capability, and (5) EfficientNet with compound scaling optimization. The experimental results are presented in Table 3.

The CNN extractor demonstrates advantages in capturing local time-frequency characteristics of vibration signals through parameter sharing and parallel computing, making it suitable for processing high-sampling-rate sensor data. However, its limited receptive field restricts long-term degradation trend modeling. Inception-ResNet employs parallel multi-scale convolutional kernels to simultaneously extract local details and global trends while mitigating partial gradient vanishing issues, albeit at the cost of increased memory consumption and computational latency due to its multi-branch architecture. The Channel Attention mechanism automatically learns optimal weights for critical frequency bands or sensor channels to suppress irrelevant information, though its effectiveness depends on the prominence of discriminative channels. EfficientNet achieves balanced model scaling across depth, width, and resolution dimensions, offering advantages for edge device deployment, but lacks explicit mechanisms for long-term degradation pattern learning.

**Figure 12:**
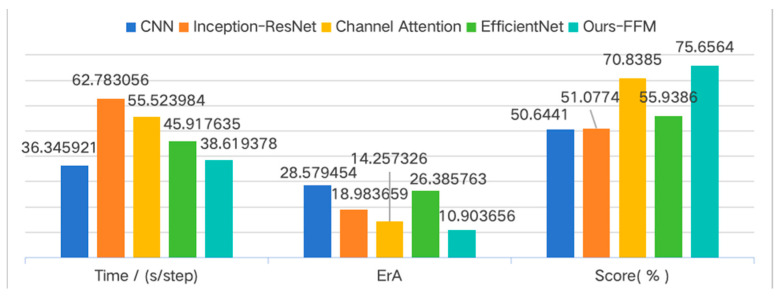
Results of comparative trials of RUL prediction using different feature extraction modules.

In contrast, the proposed FFM directly models fault evolution’s long-term dependencies through exponentially expanded receptive fields via hierarchical dilation rates, enabling comprehensive coverage of both short-term and long-term vibration patterns. Compared to architectures requiring deep layer stacking or complex branching, FFM achieves broader temporal coverage with fewer parameters, demonstrating superior suitability for real-time monitoring applications, see Figure 12.

As evidenced by the results in Table 3, the proposed FFM achieves optimal performance across all evaluation metrics, demonstrating its superior capability in extracting discriminative degradation features from rolling bearing vibration signals compared to conventional feature extraction methods.

To rigorously validate the accuracy of the proposed method, we conducted comprehensive comparisons against five established prediction approaches: (1) a Transformer-based model that captures global degradation patterns through self-attention mechanisms [36], (2) the AM-RNN attention network designed for extracting degradation features from extended historical data [37], (3) the CBAM-CNN architecture incorporating convolutional attention modules [38], (4) the DBN-RLSTM framework utilizing deep belief networks for health indicator extraction and compression [39], and (5) bearing RUL prediction network PGCN improved based on graph convolutional neural network [40]. All comparative evaluations were performed using the DAM-processed datasets (Ds1 through Ds5), with detailed results presented in Table 4.

The Transformer architecture effectively captures long-term degradation patterns in vibration signals through its self-attention mechanism, though this capability comes with significant computational overhead due to the attention matrix’s quadratic complexity and substantial memory requirements for processing lengthy sequences, while also exhibiting limited sensitivity to transient vibrational events and high-frequency noise components. In comparison, the AM-RNN framework enhances prediction accuracy by emphasizing critical timesteps through attention weighting, but its sequential processing nature inherently limits parallel computing efficiency and slows training procedures. The CBAM-CNN approach demonstrates strong performance in local time-frequency feature extraction through its channel and spatial attention mechanisms, yet struggles to effectively model temporal dependencies across extended periods. DBN-RLSTM combines the strengths of deep belief networks and recurrent architectures for multi-scale feature learning, but the resulting parameter-intensive structure presents challenges for practical deployment in resource-constrained environments. PGCN provides probabilistic life prediction intervals through graph representations, though the computational demands of sparse matrix operations in its graph convolutional layers hinder real-time application.

**Figure 13:**
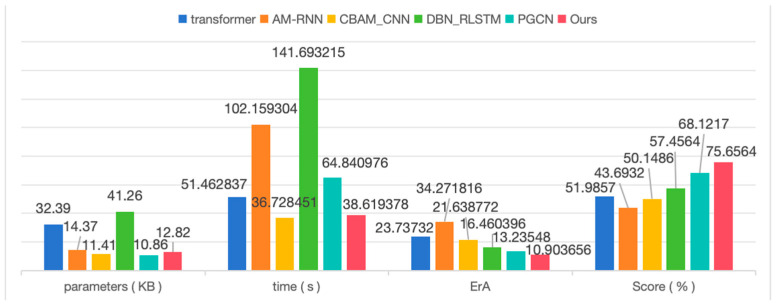
Results of comparative trials using different prediction methods.

Addressing these limitations, the proposed DCM architecture maintains temporal resolution by eliminating pooling operations while significantly reducing memory consumption and improving training stability through its exclusive reliance on dilated convolutions instead of recurrent or attention mechanisms, with the added benefit of inherent high-frequency noise suppression through carefully designed dilation intervals, all while preserving constant computational complexity regardless of input sequence length, see Figure 13.

The experimental results presented in Table 4 demonstrate the superior performance of the proposed FDBRP model across multiple evaluation dimensions. In terms of computational efficiency, the model achieves parameter complexity comparable to the most lightweight method PGCN while maintaining computational speed equivalent to the fastest approach CBAM_CNN, indicating an effective balance between model compactness and processing efficiency. More significantly, the proposed architecture establishes notable improvements in prognostic accuracy, yielding the most favorable results for both average prediction error and RUL score metrics among all compared methods.

**Figure 14:**
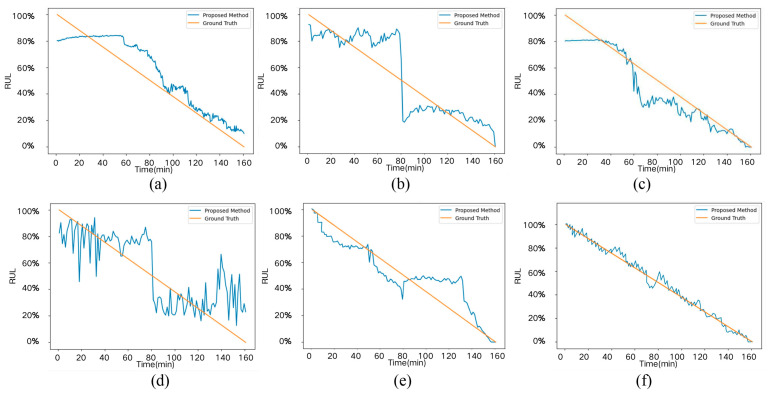
Comparison of RUL results using different prediction methods: (**a**) transformer; (**b**) AM-RNN; (**c**) CBAM_CNN; (**d**) DBN_RLSTM; (**e**) PGCN; (**f**) Ours-DCM.

As illustrated in Figure 14a–f, comparing the other five methods, the proposed FDBRP method (Figure 14f) demonstrates superior alignment with ground-truth RUL labels. This approach achieves (1) significantly reduced prediction volatility across operational periods while maintaining high accuracy, (2) precise early-stage RUL estimation approximating 100% health state, and (3) critical safety-enhanced end-of-life prediction where the method proactively converges to 0% RUL minutes before actual failure—a vital feature for risk mitigation in mechanical systems. These results collectively validate FDBRP’s advanced prognostic capability and operational practicality.

These comprehensive advancements confirm the FDBRP’s capability to simultaneously optimize computational requirements and prediction precision, particularly in capturing critical failure-stage degradation patterns essential for practical bearing life assessment applications.

### 4.4. Ablation Experiment

The FDBRP proposed in this paper consists of three integrated components: DAM for data processing, FFM for feature extraction, and DCM for life prediction. To validate the rationality of our network architecture for rolling bearing RUL prediction, we designed the following ablation experiments:

M1: A conventional CNN architecture without dilated causal mechanisms, DAM, or FFM. This baseline model represents standard convolutional neural network approaches lacking our proposed components.

M2: An enhanced CNN incorporating the DAM but still excluding FFM and dilated causal mechanisms. This configuration evaluates the standalone contribution of our data augmentation approach.

M3: An advanced CNN including both DAM and FFM, yet without implementing dilated causal convolutions. This variant assesses the combined effect of our data and feature processing components.

M4 (FDBRP): Our complete proposed model featuring DCM with dilated causal mechanisms along with both DAM and FFM. This full implementation demonstrates the synergistic performance of all integrated components.

The experimental results using dataset Ds2 are presented in Table 5. The performance improvement from M1 to M2 confirms that DAM effectively enhances the neural network’s fitting capability at the data level. The gains observed when comparing M2 with M3 demonstrate FFM’s ability to better integrate data features and optimize prediction accuracy. Finally, the superior results of M4 over M3 prove that DCM significantly improves the model’s predictive ability for bearing RUL through its dilated causal architecture.

## 5. Conclusions

This paper presents FDBRP, an integrated framework for rolling bearing remaining useful life prediction that systematically combines three key components: the Data Augmentation Module, Feature Fusion Module, and Dilated Causal Module. The methodology develops through three principal innovations:

First, DAM enhances dataset representational capacity through overlapping sliding-window sampling and multi-sensor signal fusion. By reshaping and concatenating vertical/horizontal vibration signals while implementing end-to-end life labeling (0–1 normalization), the module significantly improves neural network generalizability. Second, FFM achieves comprehensive spatiotemporal feature extraction through self-attention graph convolution for spatial relationship modeling, multi-scale temporal convolution for dynamic pattern capture, and efficient multi-scale attention that optimizes feature weighting to strengthen global-local representation consistency. Third, DCM enables parallelizable long-term temporal dependency learning through dilated causal convolutions, augmented with residual connections that regularize network training while boosting model generalization.

Within the feature extraction module, comparative analysis against state-of-the-art approaches EfficientNet, etc., confirms that our architecture maintains competitive computational efficiency while achieving reduced prediction errors. The proposed FDBRP method and advanced benchmarks PGCN, etc., were evaluated on the dataset, demonstrating that our framework establishes significant improvements in prognostic accuracy. Specifically, it yields optimal results across metrics: an average prediction error of 10.904 and an RUL score of 0.757, outperforming all comparative methods. Ablation studies further indicate a 79.96% reduction in mean error and a 64.67% enhancement in scoring performance relative to baseline models.

Experimental results confirm FDBRP’s effectiveness in accurate bearing RUL prediction. This enhanced prognostic capability enables proactive maintenance planning for rotating machinery, significantly reducing unplanned downtime and operational costs in industrial environments. Future work will investigate cross-domain prediction capability enhancements and corresponding architectural improvements to address operational condition variations, with the ultimate objective of deploying this technology for real-time health monitoring in safety-critical systems such as wind turbines and high-speed trains.

## Figures and Tables

**Table 1:** Parameters of rolling bearing LDK UER204.

Parameter Names	Value	Parameter Names	Value
Diameter of Inner Raceway	29.30 mm	Number of Balls	8
Diameter of Outer Raceway	39.80 mm	Contact Angle	0°
Bearing Circumference	34.55 mm	Basic Rated Dynamic Load	12,820 N
Ball Diameter	7.92 mm	Basic Rated Static Load	6.65 N

**Table 2:** Processing of dataset.

Experimental Dataset	Training Dataset	Test Dataset
Ds1	Bearing1, Bearing2, Bearing3, Bearing4	Bearing5
Ds2	Bearing1, Bearing2, Bearing3, Bearing5	Bearing4
Ds3	Bearing1, Bearing2, Bearing4, Bearing5	Bearing3
Ds4	Bearing1, Bearing3, Bearing4, Bearing5	Bearing2
Ds5	Bearing2, Bearing3, Bearing4, Bearing5	Bearing1

**Table 3:** Results of comparative trials of RUL prediction using different feature extraction modules.

Module	CNN	Inception-ResNet	Channel Attention	EfficientNet	Ours-FFM
Time/(s/step)	**36.345921**	62.783056	55.523984	45.917635	38.619378
ErA	28.579454	18.983659	14.257326	26.385763	**10.903656**
Score	0.506441	0.510774	0.708385	0.559386	**0.756564**

**Table 4:** Results of comparative trials using different prediction methods.

Data	Transformer		AM-RNN		CBAM_CNN		DBN_RLSTM		PGCN		Ours-DCM	
	Er_1_	Er_2_	Er_1_	Er_2_	Er_1_	Er_2_	Er_1_	Er_2_	Er_1_	Er_2_	Er_1_	Er_2_
Ds_1_	24.3276	14.8765	51.0434	23.7797	26.0963	33.9878	14.3276	13.4728	25.9747	16.0576	11.4885	10.8724
Ds_2_	12.8765	24.3276	50.8112	23.6769	15.5812	22.3487	16.4821	15.8234	11.5913	12.5977	8.9911	6.2172
Ds_3_	23.4821	29.4821	50.1597	23.1725	28.9332	28.6946	25.9461	20.7589	10.5796	10.5424	11.1662	8.3452
Ds_4_	30.9461	23.6543	50.2571	23.2184	17.6004	20.845	9.8765	14.6391	15.9226	13.4653	11.1667	12.3306
Ds_5_	27.6543	25.9461	50.5282	23.2179	13.2471	6.8082	17.6543	16.2847	10.4042	7.9844	14.8619	14.6488
Para.	32.39 KB		14.37 KB		11.41 KB		41.26 KB		**10.86 KB**		12.82 KB	
time	51.462837 s		102.159304 s		**36.728451 s**		141.693215 s		64.840976 s		38.619378 s	
$\overset{̿}{{Er}_{A}}$	23.73732		34.271816		21.638772		16.460396		13.23548		**10.903656**	
$\bar{Score}$	0.519857		0.436932		0.501486		0.574564		0.681217		**0.756564**	

**Table 5:** Results of ablation experiments.

Model	M1	M2	M3	M4
Er_A_	36.658306	33.586026	18.467293	**7.326761**
Score	0.438725	0.504729	0.668674	**0.722447**

## Data Availability

The data used in this study are openly available in a publicly accessible repository, and the corresponding DOI is as follows: DOI: 10.3901/JME.2019.16.001.

## Abbreviations

The following abbreviations are used in this manuscript:

RUL	Remaining Useful Life
PHM	Prognostics and Health Management
EMD	Empirical Mode Decomposition
VMD	Variational Mode Decomposition
CNN	Convolutional Neural Network
FCN	Fully Convolutional Network
RNN	Recurrent Neural Networks
Bi-LSTM	Bidirectional LSTM
DRU	Gated Recurrent Unit
ResNet	Residual Connections
TCN	Temporal Convolutional Network
CAM	Convolutional Attention Module
DRN	Deep Residual Networks
SC	shortcut connections
DAM	Data Augmentation Module
DCM	Dilated Causal Module
FFM	Feature Fusion Module
AGM	Self-attention-graph Convolution module
MTM	Multi-scale Time Convolution module
EMA	Efficient Multi-scale Attention module
MPTCN	Multiple Parallel Temporal Convolutional Network
FDBRP	Feature fusion and Dilated causal convolution model for Bearing Remaining useful life Prediction
MAE	Mean Absolute Error
RMSE	Root Mean Square Error
MAE_sh	Mean Absolute Error in the second half of the stage
RMSE_sh	Root Mean Square Error in the second half of the stage
